# MicroRNA 603 acts as a tumor suppressor and inhibits triple-negative breast cancer tumorigenesis by targeting elongation factor 2 kinase

**DOI:** 10.18632/oncotarget.14264

**Published:** 2016-12-27

**Authors:** Recep Bayraktar, Martin Pichler, Pinar Kanlikilicer, Cristina Ivan, Emine Bayraktar, Nermin Kahraman, Burcu Aslan, Serpil Oguztuzun, Mustafa Ulasli, Ahmet Arslan, George Calin, Gabriel Lopez-Berestein, Bulent Ozpolat

**Affiliations:** ^1^ Department of Experimental Therapeutics, The University of Texas MD Anderson Cancer Center, Houston, Texas, USA; ^2^ Department of Medical Biology, School of Medicine, Gaziantep University, Gaziantep, Turkey; ^3^ Department of Biology, Kirikkale University, Kirikkale, Turkey; ^4^ Center for RNA Interference and Non-Coding RNAs, The University of Texas MD Anderson Cancer Center, Houston, Texas, USA

**Keywords:** eEF2K, triple negative breast cancer, liposomes, nanoparticles, miR-603

## Abstract

Triple negative breast cancer (TNBC) is an aggressive type of breast cancer characterized by the absence of defined molecular targets, including estrogen receptor (ER), progesterone receptor (PR), human epidermal growth factor receptor 2 (HER2) and is associated with high rates of relapse and distant metastasis despite surgery and adjuvant chemotherapy. The lack of effective targeted therapies for TNBC represents an unmet therapeutic challenge. Eukaryotic elongation factor 2 kinase (eEF2K) is an atypical calcium/calmodulin-dependent serine/threonine kinase that promotes TNBC tumorigenesis, progression, and drug resistance, representing a potential novel molecular target. However, the mechanisms regulating eEF2K expression are unknown. Here, we report that eEF2K protein expression is highly up-regulated in TNBC cells and patient tumors and it is associated with poor patient survival and clinical outcome. We found that loss/reduced expression of miR-603 leads to eEF2K overexpression in TNBC cell lines. Its expression results in inhibition of eEF2K by directly targeting the 3-UTR and the inhibition of tumor cell growth, migration and invasion in TNBC. *In vivo* therapeutic gene delivery of miR-603 into TNBC xenograft mouse models by systemic administration of miR-603-nanoparticles led to a significant inhibition of eEF2K expression and tumor growth, which was associated with decreased activity of the downstream targets of eEF2K, including Src, Akt, cyclin D1 and c-myc. Our findings suggest that miR-603 functions as a tumor suppressor and loss of miR-603 expression leads to increase in eEF2K expression and contributes to the growth, invasion, and progression of TNBC. Taken together, our data suggest that miR-603-based gene therapy is a potential strategy against TNBC.

## INTRODUCTION

Breast cancer (BC) is the most common malignancy among women and the second leading cause of cancer-related deaths worldwide [1]. Every year in the United States, nearly 232,000 cases of invasive breast cancer and 65,000 cases of *in situ* breast cancer are diagnosed, and more than 40,000 women die to breast cancer [2]. BC is a highly complex and heterogeneous disease with distinct biological and clinical behaviors [3]. BC is classified into five major subtypes according to molecular features and intrinsic expression of the genes encoding the estrogen receptor (ER), progesterone receptor (PR), and human epidermal growth factor receptor 2 (HER2): luminal A (ER and/or PR positive and HER2 negative), luminal B (ER or PR positive and HER2 positive), HER2 overexpressing, normal-breast like and basal-like or triple-negative breast cancer (TNBC) phenotype [3].

TNBC accounts for approximately 10-20% of all cases of breast cancer and is characterized by the absence of yet defined molecular targets, including estrogen receptor (ER), progesterone receptor (PR) and human epidermal growth factor receptor 2 (HER2) [4, 5]. Thus, therapies targeting ER (i.e., tamoxifen) and HER2 (also known as eERB2) (i.e., trastuzumab) are ineffective against TNBC [5]. The other important characteristics of TNBC include aggressive clinical behavior, early relapses, and metastasis as well as reduced sensitivity to conventional therapies. The poor clinical outcome and short overall patient survival predominantly attributed to intratumoral heterogeneity and mutated TP53, which is detected in up to 84% of TNBC cases [5–7]. A better understanding of the biology of TNBC and the underlying molecular mechanisms are needed to identify novel therapeutic targets and develop highly effective targeted therapies for improved patient outcomes [8–10].

Recently, emerging evidence has revealed that eukaryotic elongation factor 2 kinase (eEF2K) is a potential molecular driver in several cancers, including pancreatic, brain and breast cancer [11–16]. eEF2K is the only calcium/calmodulin activated member of the α-kinase family and is considered an atypical kinase since its catalytic domain is not structurally similar to those of conventional protein kinases [17, 18]. eEF2K activity is regulated by multiple mechanisms to control the rate of protein chain elongation by phosphorylating/inactivating eEF2 (at threonine 56), which mediates the movement of the ribosome on transfer RNA (tRNA) from the A site to the P site [19–23]. eEF2K promotes cell survival in conditions of nutrient deprivation, hypoxia and metabolic stress by regulating the rate of translation [24]. Recently, eEF2K was shown to promote cell proliferation, cell migration, invasion, epithelial-mesenchymal transition (EMT) and TNBC tumorigenesis and progression through modulating the cell cycle (G1/S transition) by regulating cyclin D1, c-myc, PI3K/Akt, Src/Fak and insulin-like growth factor receptor (IGFR) signaling [11, 13, 14, 16]. Therapeutic targeting of eEF2K triggers apoptosis and suppresses TNBC tumor growth, in addition to and increased doxorubicin and paclitaxel efficacy [16]. These reports suggest that eEF2K is a critical factor for breast cancer progression and the strategies aimed at manipulating the activity of eEF2K may aid the development of novel treatment regimens for TNBC.

Non-coding RNAs such as microRNAs (miRNA) have emerged as new regulators of gene expression across various biological processes, including cell cycle regulation, differentiation, metabolism and aging. miRNAs are involved in many diseases such as cardiovascular and neurodegenerative diseases and but they also play an a vital role in the pathogenesis of human cancers and clinical applications as therapeutics [25]. miRNAs can be used to classify a wide variety of human cancers with particular signatures and define the molecular architecture of human cancers [26–30]. miRNAs are 18-25 nucleotides in length and regulate the expression of target genes at the posttranscriptional level through interaction with complementary sequences usually found in the 3’-UTRs of target mRNAs, resulting in inhibition of translation and/or in mRNA degradation [25].

Several oncogenic and tumor suppressor miRNAs have been identified as the promoters of tumor formation and growth when aberrantly expressed in various cancers [27]. Oncogenic miRs are frequently over-expressed in cancer tissues, including miR-21, miR-17-92, miR-155 and miR-372, while miRs such as miR-34 and the *let-7* family miR-15a and miR-16-1 are considered as tumor suppressors and their expression is often reduced in cancer tissues [27]. Over the years of studies, growing evidences have shown that miRNAs have an important role in breast cancer progression, invasion, angiogenesis and metastasis. Apparently, some miRNAs functionally take part in several important cell proliferation pathways such Src, and MAPK and the aberrant expression of these miRNAs is responsible for evading growth suppressors and sustaining proliferative signaling in breast cancer cells [31–33]. miRNA 603 (miR-603) was recently identified through genome-wide analysis in glioblastoma [34] and thyroid cancer with a potential role in cell transformation [35]. However the role of miR-603 in breast cancer has not been characterized previously and was the focus of the current study.

This study demonstrates for the first time that miR-603 functions as tumor suppressor in TNBC and its expression plays an important role in TNBC cell proliferation, migration/invasion, and tumorigenesis through the regulation of eEF2K. Our data also suggest that miR-603-based gene therapy or eEF2K targeted approaches may be a potential therapeutic strategy against TNBC.

## RESULTS

### eEF2K expression is upregulated in TNBC cell lines and patient tumors

**Figure 1:**
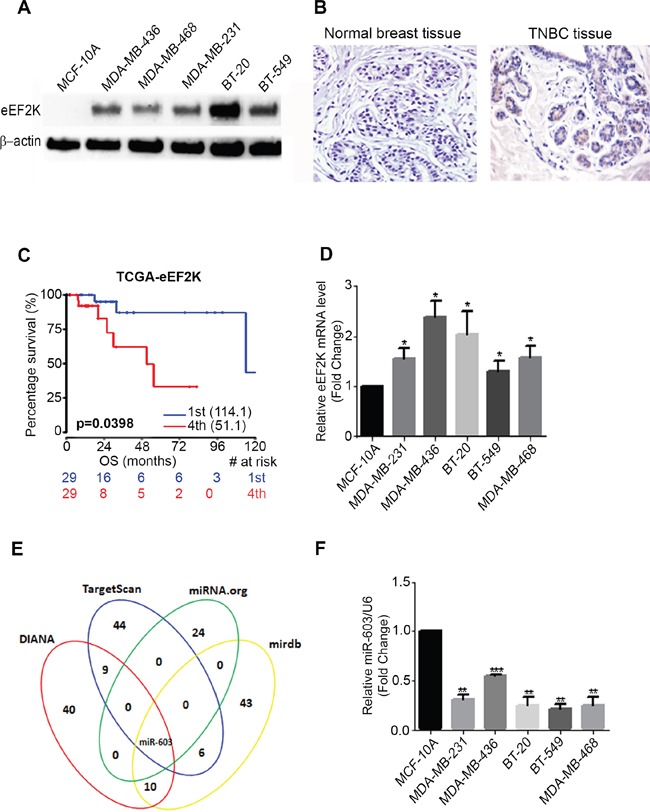
eEF2K protein and mRNA is overexpressed in TNBC cell lines and patients tumor samples **A**. eEF2K protein expression levels in TNBC cell lines were higher than in the normal breast epithelial cells (MCF-10A). **B**. The expression levels of eEF2K in patient tumor tissues and adjacent normal tissues were determined by immunohistochemistry **C**. High protein expression of eEF2K was associated with poor overall survival in breast cancers patients with low and high eEF2K expression, log-rank *p* = 0.0398) as determined by Kaplan-Meier analysis. **D**. Relative expression levels of eEF2K mRNA in TNBC cell lines were analyzed with qPCR. **E**. The algorithms including TargetScan, miRDB, Diana microT, and microRNA predict that the 3’-UTR of eEF2K is targeted by miR-603. **F**. Relative expression levels of miR-603 in TNBC cell lines and in MCF-10A cells were quantified by qPCR using specific primers. The data were normalized to the expression of U6 small nuclear RNA and are shown as means with SDs for three independent experiments.

To evaluate eEF2K expression in TNBC we determined the protein expression level of eEF2K in TNBC cell lines and patient tumors by Western blot and immunohistochemistry, respectively. The expression levels of eEF2K protein were significantly higher in MDA-MB-436, MDA-MB-468, MDA-MB-231, BT-20, and BT-549 cells than in the non-tumorigenic normal breast cell epithelium MCF-10A cells (Figure 1A). We also investigated eEF2K expression in 9 TNBC patient and 10 normal breast tissue biopsy samples. The expression levels of eEF2K protein were highly positive in TNBC patient biopsy samples (66.66% 6 and 9 patient tumors) than in normal breast tissues (Figure 1B and Supplementary Figure 1A).

### eEF2K expression is associated with poor prognosis and breast cancer patient survival

To elucidate the clinical significance of eEF2K protein expression, we first analyzed TCGA database and determined the prognostic value of eEF2K in 58 breast cancer patients with basal subtype. Overall survival curves were plotted according to eEF2K gene expression levels using the Kaplan-Meier method. Overall survival rate was significantly lower in patients with high eEF2K expression than in patients with low eEF2K expression (*p* = 0.0398) (Figure 1C).

### miR-603 expression in TNBC is inversely correlated with eEF2K mRNA expression

We first analyzed the levels of eEF2K mRNA in TNBC cell lines, including MDA-MB-231, MDA-MB-436, BT-20, BT-549 and MDA-MB-468 cell lines and the non-tumorigenic cell line MCF-10A via qPCR. The results showed that eEF2K mRNA levels were upregulated in TNBC cell lines (Figure 1D). To identify miRNAs that target and regulate eEF2K gene expression, we used four different algorithms that predict the mRNA targets of miRNAs: TargetScan (http://www.targetscan.org/cgi-bin/targetscan/vert_70), miRDB (http://mirdb.org/cgi-bin/search.cgi), microRNA.org (http://www.microrna.org/microrna/searchGenes.do), and Diana microT (http://diana.imis.athena-innovation.gr/DianaTools/) to select miRNAs that may target eEF2K. Based on this target prediction strategy, miR-603 was the common miRNA that targets eEF2K in all four database (Figure 1E). We also selected miR-3613-3p and miR-3163 that were common in the three of the databases for binding scores for targeting eEF2K (Figure 1E).

The basal expression levels of miR-603, miR-3613-3p, and miR-3163 in TNBC cell lines (MDA-MB-231, MDA-MB-436, and BT-20) were compared to their expression levels in MCF-10A cells by qPCR. Only miR-603 expression was lower in all three TNBC cell lines than in MCF-10A cells (Figure 1F), suggesting an inverse relationship of miR-603 to eEF2K and potential role for miR-603 directly binding eEF2K mRNA and regulating its expression. The basal expression levels of miR-3613-3p and miR-3163 did not show any correlation with eEF2K expression in TNBC cell lines (Supplementary Figure 1B and 1C). Therefore, we hypothesized based on target prediction tools and the inverse correlation that eEF2K is a direct target of miR-603 in human TNBC cells.

### miR-603 expression suppresses eEF2K in TNBC cells

**Figure 2:**
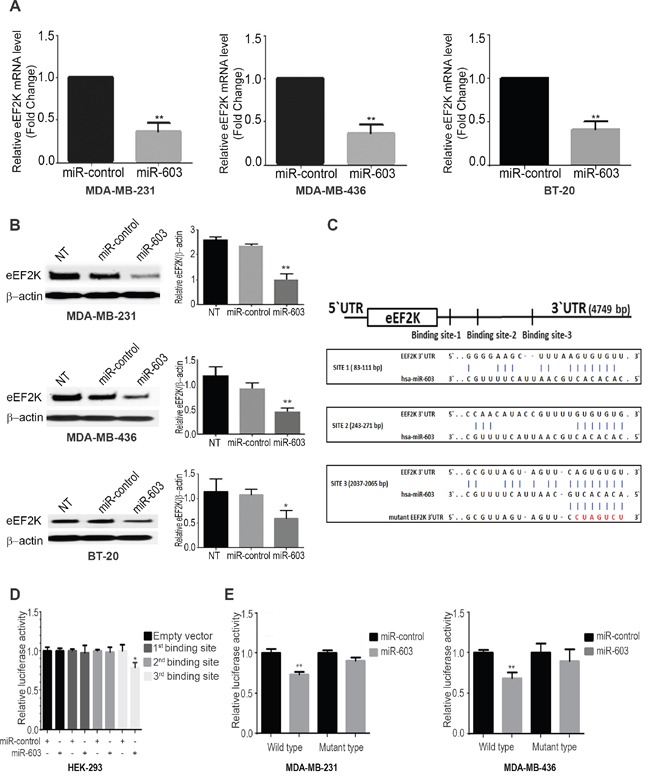
miR-603 negatively regulates eEF2K expression levels in TNBC cells by directly binding to the eEF2K 3’-UTR **A**. miR-603 expression leads to decreased eEF2K mRNA expression levels in MDA-MB-231, MDA-MB-436 and BT-20 in TNBC cells. Cells lines were analyzed for eEF2K mRNA levels by qPCR 48 h after miR-603 transfection. **B**. miR-603 expression decreases eEF2K protein expression levels in MDA-MB-231, MDA-MB-436 and BT-20 cells. TNBC cells were transfected with the miR-603 mimic or miR-control, and eEF2K protein levels were analyzed by Western blotting 72 h after transfection. B-Actin was used as a loading control. Band intensities were quantified using densitometric analysis (right panel, NT, not transfected. **C**. Three predicted binding sites of miR-603 in the 3’-UTR of human wild-type eEF2K and their sequences. Mutations in the seed sequence of the full-length eEF2K 3’-UTR are shown in red. **D, E**. Luciferase reporter assay showed that miR-603 directly targets the eEF2K 3’-UTR-luciferase reporter (wild type or mutant miR-603 binding sides), in HEK-293, MDA-MB-231 and MDA-MB-436 cells incubated with the miR-603 mimic for 48 h before analysis. The firefly luciferase activity of the reporter was normalized to the internal Renilla luciferase activity. The data are presented as means with SDs for three independent experiments. *p < 0.05.

Since miR-603 is markedly downregulated in TNBC cell lines, we ectopically overexpressed miR-603 in MDA-MB-231, MDA-MB-436, and BT-20 cells and measured changes in the expression of eEF2K mRNA and protein levels. As shown in Supplementary Figure 2A, in miR-603 mimic-transfected MDA-MB-231, MDA-MB-436, and BT-20 cells, miR-603 was expressed at levels that were 18, 5.1, and 14 times higher, respectively, than in the corresponding cell lines transfected with the control miRNA. Ectopic overexpression of miR-603 significantly suppressed the eEF2K mRNA levels in MDA-MB-231, MDA-MB-436 cells and BT-20 cells (Figure 2A). We further demonstrated by western blot that miR-603 expression also led to reduced eEF2K protein expression in MDA-MB-231 (59.8% reduction), MDA-MB-436 (47.6% reduction) and BT-20 cells (46.8% reduction) (Figure 2B), indicating that miR-603 is a potential regulator of eEF2K expression in TNBC cells.

### miR-603 regulates eEF2K mRNA expression by directly binding to 3’-UTR region

To determine whether the negative regulatory effect of miR-603 on eEF2K expression is mediated through direct binding to the predicted sites in the 3’-UTR of the eEF2K mRNA according to four different algorithms, we evaluated three different predicted miR-603 binding sites were found in3’-UTR region of eEF2K gene (Figure 2C). The resulting plasmids were transfected into HEK293 cells along with the miR-603 mimic or the scrambled negative-control miRNA. As shown in Figure 2D, luciferase activity was significantly reduced in cells transfected with the plasmid containing the binding site 3 in the 3’-UTR of miR-603 (p = 0.03). To further provide proof of the binding of miR-603 to the specific binding motifs, we also introduced point mutations (CACTGCC->TATGACT) into the corresponding miR-603 binding site in the 3’-UTR of eEF2K. pEZX-MT06 miRNA reporter vector containing one point mutation was also used. Only the mutation in binding site 3 (2037–2065 bp; Figure 2C) completely reversed the effect of overexpression of miR-603 (Supplementary Figure 2B). We also performed miRNA luciferase reporter assay in MDA-MB-436 and MDA-MB-231 cells resulting in luciferase activity was significantly reduced in cells transfected with the plasmid containing the binding site 3 in the 3’-UTR of miR-603 (MDA-MB-436; p < 0.005 and MDA-MB-231; p<0.05). Collectively, our findings suggest that binding site 3 contributes to the interaction between miR-603 and eEF2K mRNA and that miR-603 directly recognizes and binds to the eEF2K 3’-UTR to regulate the expression of the eEF2K mRNA transcript.

### miR-603 suppresses proliferation and clonogenicity of TNBC cells by regulating signaling pathways involved in cell proliferation, survival and invasion

**Figure 3:**
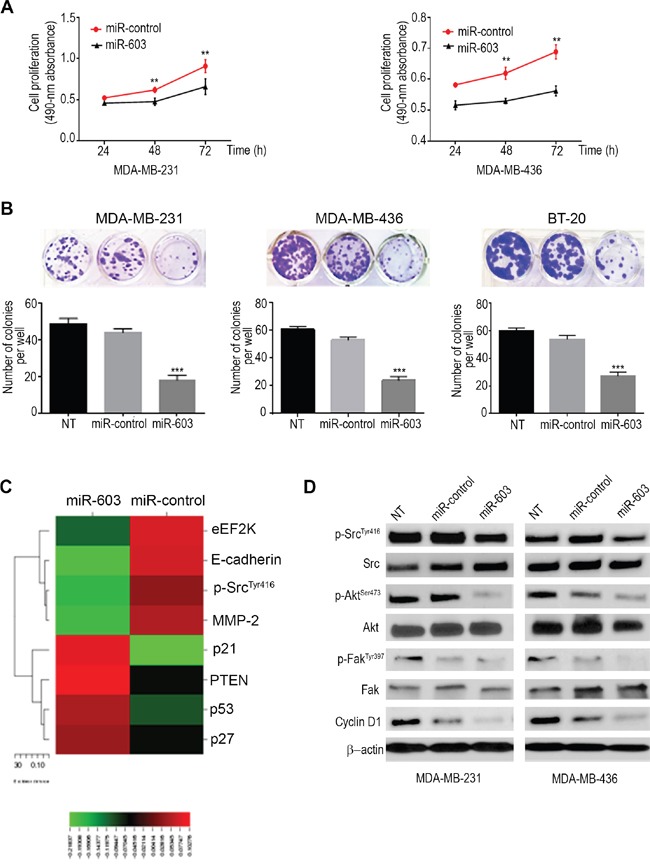
Ectopic expression of miR-603 suppresses TNBC cell proliferation and clonogenic ability *in vitro* **A**. The short term effects of ectopic expression of miR-603 on the proliferation of MDA-MB-231 and MDA-MB-436 cells examined by cell proliferation assay (MTS assay) are shown as growth curves. The data are means ± SDs (*p <0.05). **B**. Effects of overexpression of miR-603 on the colony formation or clonogenic ability of MDA-MB-231, MDA-MB-436 and BT-20 cells. Upper panels, representative culture dishes from a colony formation assay. A lower panel, the number of colonies formed was normalized to the number of colonies formed by the control miRNA (miR-control)-transfected cells. The data are means ± SDs. ***p < 0.001. **C**. A heat map of RPPA results revealed an array of altered proteins in MDA-MB-231 cells. The expression ratios for a given sample group of interest were represented by their mean. Rows, proteins; columns, signal ratios of miR-603- or control miRNA-transfected MDA-MB-231 cells. For each protein, the red color indicates that the expression level of that protein was higher in miR-603-transfected cells than in control miRNA-transfected cells, and the green color indicates that the expression level was lower. **D**.Western blot analysis of p-EF2 ^Thr56^, p-Src ^Tyr416^, total Src, p-Fak^Tyr397^, total Fak, p-Akt ^Ser473^, total Akt and cyclinD1 in the indicated cells after 72-h of transfection. β-Actin was used as a loading control.

To examine the short term effects (72h) of miR-603 on proliferation of TNBC cells, we used MDA-MB-231 and MDA-MB-436 cells and performed proliferation (MTS) assay. The results showed that in miR-overexpressing MDA-MB-231 and MDA-MB-436 cells, absorbance at 490 nm was 27.40 and 18.34 units lower, respectively, than in the corresponding control cells (72 h) (Figure 3A).

We further examined the effects of miR-603 on TNBC cell proliferation and clonogenicity using a colony formation assay. The miR-603-transfected MDA-MB-231, MDA-MB-436 and BT-20 cells formed fewer (18.00 ± 2.64, 24.00 ± 2.64 and 27.00± 1.73 colonies per well, respectively) and smaller colonies than the corresponding control-mimic transfected cells (44.00 ± 2.00, 53.00 ± 2.00 and 53.67 ± 1.76 colonies per well, respectively) (Figure 3B). A significant decrease in cell proliferation and colony formation was observed in MDA-MB-231 (***p=0.0003), MDA-MB-436 (***p=0.0002) and BT-20 (****p=0.0004) cells transfected with miR-603. These results confirm the growth inhibiting effects of miR-603 in TNBC cells. The miR-603-inhibitor-transfected MDA-MB-231 cells did not generate significantly different number of colonies (44.00 ± 1.15 colonies per well) compared to the corresponding control cells (44.67 ± 3.48 colonies per well). Down regulation of the expression of miR-603 with inhibitors had no effect on the growth of MDA-MB-231 cells (Supplementary Figure 3A) probably because the endogenous levels of miR-603 in these cells are relatively low.

To elucidate the effects of miR-603 expression on downstream targets/signaling pathways in MDA-MB-231 cells, we used RPPA to analyze MDA-MB-231 cells transfected with miR-603 or the control miRNA (Figure 3C). RPPA results indicated that ectopic expression of miR-603 decreased the expression of eEF2K, p-Src, matrix metalloproteinase 2 (MMP2), and E-cadherin but increased the expression of p21, PTEN, p53, and p27 in MDA-MB-231 cells. These results revealed that overexpression of miR-603 may alter pathways that are involved in cell proliferation, survival, migration, and invasion, the cell cycle, and/or apoptosis.

To evaluate that the activity of Akt and Src is affected by the miR-603/eEF2K axis, MDA-MB-231 and MDA-MB-436 cells were transfected with miR-603 or the control miRNA. Western blot analysis showed reduced levels of p-Akt (Ser473), cyclin D1, p-Src (Tyr416) and p-Fak (Tyr397) following miR-603 ectopic overexpression in MDA-MB-231 and MDA-MB-436 cell lines (Figure 3D).

### miR-603 expression impairs TNBC cell motility, migration and invasion by downregulation of eEF2K

**Figure 4:**
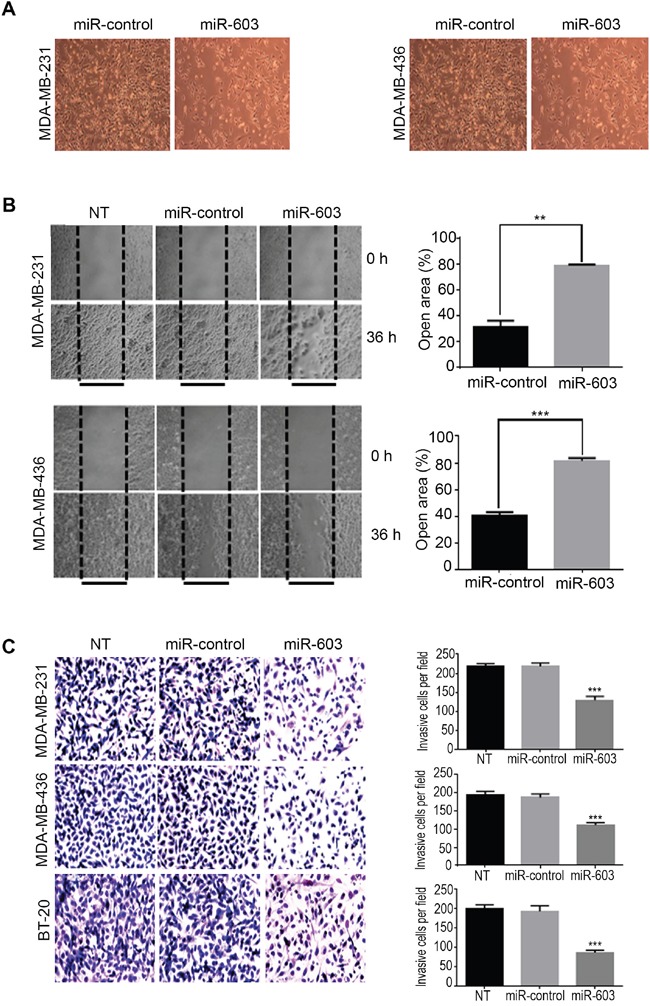
Transfection of TNBC cells with miR-603 suppresses migration and invasion of the cells *in vitro* **A**. Morphological changes in MDA-MB-231 and MDA-MB-436 cells after 48-h transfection with 50 nM miR-603 or control miRNA. Representative phase contrast micrographs are shown. **B**. MDA-MB-231 and MDA-MB-436 cell lines that were transfected with miR-603, or miR-control or that did not undergo transfection (NT) were assessed for migration with the wound healing assay. After 72-h transfection, a wound was formed by scraping, and the area of the wound was measured at 0 and 36 h. The relative percentages of wound closure per field are shown on the right as means ± SDs. **C**. the invasiveness of MDA-MB-231, MDA-MB-436 and BT-20 cells was assessed by using a matrigel transwell assay. The cells were transfected with miR-603 or miR-control or not treated (NT). After 72-h transfection, the cells were transferred to transwell chambers and incubated for 24 h. The invading cells were counted, and mean ± SDs from triplicate experiments are shown on the right (***p < 0.001).

After finding miR-603 inhibits Src/Fak signaling axis, next, we investigated whether miR-603 expression suppresses tumor cell migration and invasion, we first transfected MDA-MB-231 and MDA-MB-436 cells with miR-603. Figure 4A shows the effects of miR-603 expression on cell migration and the associated morphological changes in the two cell lines. After 24 h, the control miRNA-transfected cells covered 65% to 85% of the space generated by physically removing cells, whereas miR-603-overexpressing cells covered less than 40% of the space (Figure 4B), indicating that miR-603-expression leads to reduced cell motility and migration.

To examine the role of miR-603 expression in regulating the ability of TNBC cell invasion, we performed *in vitro* matrigel invasion assay. The results showed that the number of cells invading matrigel was significantly lower in miR-603-transfected cells than in control miR transfected cells (MDA-MB-231, 111.8 ± 2.469 vs. 187.0 ± 3.967 cells, *p* < 0.001); MDA-MB-436, 129.2 ± 4.206 vs. 218.0 ± 3.795 cells, *p* < 0.001 and BT-20, 86.67 ± 2.472 vs. 192.8 ± 5.952 cells (*p* < 0.001) (Figure 4C). Together, these results suggest that miR-603 expression suppresses migration and invasion of TNBC cells.

### eEF2K inhibits cell proliferation and migration/invasion of TNBC cells

**Figure 5:**
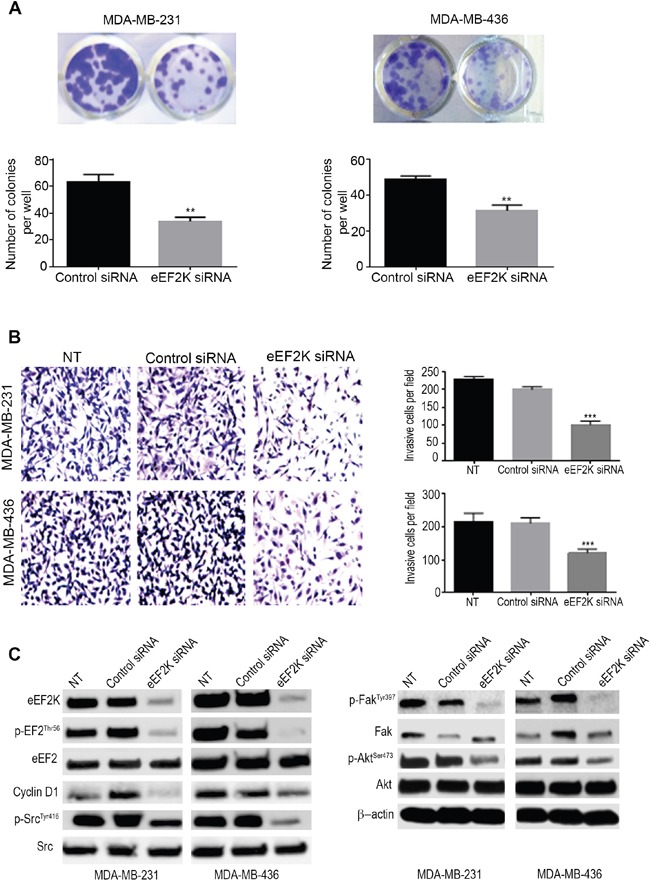
Knockdown of eEF2K by siRNA leads to inhibition of cell clonogenicity, migration, and invasion *in vitro* **A**. The silencing of eEF2K by siRNA (50 nM) significantly reduced the number of colonies formed by MDA-MB-231 and MDA-MB-436 cells (***p < 0.001). Cells were transfected every 4 days with the control or eEF2K siRNA. **B**. MDA-MB-231 and MDA-MB-436 cell invasion was assessed by using a matrigel transwell assay. Cells were transfected with eEF2K siRNA or control siRNA or not transfected (NT)); after 72-h transfection, the cells were transferred to transwell and incubated for 24 h and cells invading matrigel and passing through the membrane we counted by light microscope. **C**. Western blot analysis of p-EF2^Thr56^, p-Src^Tyr416^, total Src, p-Fak^Tyr397^, total Fak, p-Akt^Ser473^, total Akt and cyclin D1 in the indicated cells. β-Actin was used as a loading control.

To further elucidate the mechanism by which miR-603 regulates cell proliferation, motility and invasion through eEF2K inhibition in TNBC cells, we investigated the effects of eEF2K knockdown on proliferation, migration, and invasion of TNBC cells. MDA-MB-231 and MDA-MB-436 cells transfected with eEF2K siRNA had significantly lower colony formation ability (34.00 ± 1.73; and 31.33 ± 1.85 colonies per well, respectively) than control cells (63.33 ± 3.18 and 48.67 ± 1.20 colonies per well, respectively)(**p=0.0035; **p=0.0026) (Figure 5A). These results confirm the growth inhibiting effects of silencing of eEF2K in TNBC cells. Additionally, knockdown of eEF2K significantly inhibited *in vitro* cell invasion as indicated by reduced the number of matrigel invading MDA-MB-231 and MDA-MB-436 cells (Figure 5B). The results showed that the number of cells invading was significantly lower in eEF2K-transfected cells than in control siRNA transfected cells (MDA-MB-231, 102.2 ± 3.95 vs. 199.5 ± 2.97 cells, *** *p* < 0.0001) and MDA-MB-436, 121.7 ± 4.50 vs. 211.2 ± 6.60 cells, ***p < 0.0001 (Figure 5B). These results indicate that the silencing of eEF2K inhibits proliferation and invasion of TNBC cells and recapitulates the effects of miR603, indicating that the miR-603-induced effects are mediated by inhibition of eEF2K.

Furthermore, knockdown of eEF2K by siRNA markedly reduced the expression levels of p-EF2, p-Src, p-Akt, p-Fak, and cyclin D1 but not total EF2, Src, Akt, and Fak in MDA-MB-231 and MDA-MB-436 cells compared to the levels in cells treated with the control siRNA (Figure 5C). These results showed that suppression of Akt and Src by miR-603 is accompanied by reduced TNBC cell proliferation and survival, indicating that miR-603 recapitulates the effects of eEF2K knockdown in TNBC cells.

Given the suggested contribution of eEF2K signaling inhibition through miR-603 in TNBC cells, we determined whether overexpression of eEF2K gene could reverse the effect of miR-603-mediated inhibition. Thus, we overexpressed human eEF2K gene in MDA-MB-231 cells, (see in Experimental Procedures), and the levels of eEF2K and pEF2 expression were confirmed by Western Blotting (Supplementary Figure 3B). We further examined the effect of miR-603 mimic or miR-control transfections in eEF2K overexpressed or empty vector containing MDA-MB-231 cells. As a result, eEF2K overexpression reversed the effects of miR-603 mimic in MDA-MB-231 cells and led to reduced inhibition in the expression of eEF2K and its downstream targets such as p-EF2, p-Src, and p-Fak, indicating that the effect of miR-603 on key signaling pathways/molecules is mediated by eEF2K downregulation (Supplementary Figure 3C).

### *In vivo* gene therapy by systemic administration of miR-603 reduces growth of orthotopic TNBC xenograft tumors in mice

**Figure 6:**
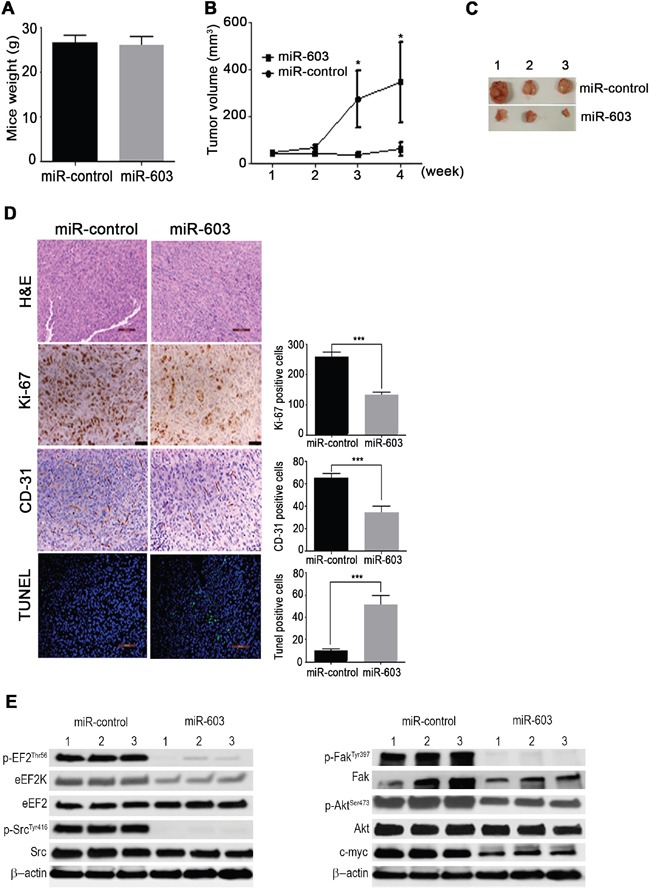
*In vivo* systemic administration of miR-603 nanoparticles inhibits growth TNBC xenografts in mice **A, B, C**. MDA-MB-436 cells were orthotopically injected into the mammary fat pad of female athymic nude mice. The mice were then treated with either nanoliposomal control miRNA (miR-control) or miR-603 nanoparticles (0.3 mg/kg [8 μg/mouse] intravenously twice per week for 4 weeks; five mice per group). Tumor volumes were measured weekly and are shown as means ± SDs. miR-603 treatment did not cause any change in the mean mouse weight after 4 weeks. **D**. Samples of MDA-MB-436 tumors from the control and miR-603-treated mice were stained with hematoxylin and eosin. Scale bar = 100 μm. (D) Tumor cell proliferation and micro vessel density were analyzed by evaluating the expression of Ki-67 and CD-31, respectively, in tumor tissues by immunohistochemistry; *D*, *In vivo* induction of apoptosis was analyzed by TUNEL assay in MDA-MB-436 tumor xenografts. Magnification,×20. **E**. Western blot analysis demonstrated that silencing of eEF2K in MDA-MB-436 tumor samples reduced eEF2K, p-eEF2, p-Src, p-Akt, c-myc and p-Fak protein expression levels. β-Actin was used as a loading control.

To determine the role of miR-603 in TNBC tumorigenesis and progression as well as the therapeutic potential of miR-603-based gene therapy, we assessed the effects of miR-603 expression in a MDA-MB-436 orthotopic xenograft mouse model. MDA-MB-436 cells were orthotopically implanted into the right mammary fat pad of nude mice. About 2 weeks later, liposomal nanoparticles incorporating miR-603 or the controls (0.3 mg/kg) were intravenously (i.v) administered once a week. No toxic effects were observed in the 4 weeks after the administration of pegylated liposomal miR-603 nanoparticles; the mice appeared healthy and did not lose weight during the 4-weeks of treatment (Figure 6A). The mean weights of mice treated with miR-603 and mice treated with the control miRNA were 26.5 ± 0.8 g and 25.9 ± 0.85 g, respectively (Figure 6A). The volumes of xenograft tumors were measured every week for 4 weeks (Figure 6B). Four weeks after the treatment, the xenograft tumors were excised and analyzed for eEF2K down modulation, cell proliferation, apoptosis, and angiogenesis as well as pathway regulation. The mice treated with liposomal miR-603 had a significantly lower rate of tumor growth and drastically smaller tumors than the control mice (Figure 6B, 6C).

### *In vivo* delivery of miR-603 inhibits cell proliferation and microvessel density and induces apoptosis in TNBC tumors

The proliferative activity of the MDA-MB-436 tumor cells was measured by evaluating the expression of the proliferation marker Ki-67 by immunohistochemistry. The number of Ki-67-positive tumor cells was significantly lower in miR-603-treated mice than in the control mice (*p* < 0.001) (Figure 6D). Immunohistochemical analyses for microvessel density showed that the number of CD31-positive cells was dramatically lower in the miR-603-treated group than in the control group (Figure 6D). We also assessed the rate of apoptosis using the TUNEL assay (Figure 6D) and found that the miR-603 treatment resulted in a significantly higher number of TUNEL-positive cells than did treatment with the control miRNA, indicating induction of apoptosis (*p* < 0.001). Taken together, our results revealed that miR-603 inhibited TNBC tumor growth in association with significant inhibition of tumor cell proliferation and angiogenesis and induction of apoptosis.

### *In vivo* delivery of miR-603 inhibits eEF2K, Akt, Src/Akt and c-myc in TNBC tumors

Tumor samples were further analyzed by western blotting for the expression of eEF2K and the downstream targets of eEF2K, including p-EF2, p-Src, p-Akt, p-Fak, and c-myc [16]. The results showed that the expression levels of eEF2K, p-EF2, p-Src, p-Akt, p-Fak, and c-myc were markedly lower in the miR-603-treated group than in the control miRNA-treated group (Figure 6E). Overall, the data suggest that miR-603 treatment effectively suppressed the expression of eEF2K protein in tumors.

## DISCUSSION

In this study, we report for the first time that miR-603 acts as a tumor suppressor in TNBC by directly regulating eEF2K expression, thereby inhibiting cell proliferation, survival, invasion, and tumorigenesis. We also demonstrated that eEF2K is highly overexpressed in TNBC cells and reduced or loss of miR-603 expression contributes to upregulation of eEF2K and TNBC tumor growth and progression.

We previously reported that EF2K signaling is one of the critical drivers of TNBC tumorigenesis and its inhibition suppresses tumor growth and significantly enhances the efficacy of most commonly used chemotherapeutics in TNBC tumor models [16]. The current study indicated that the majority of TNBC patient tumors (66.6%) are strongly positive for eEF2K expression by IHC. Our study also demonstrated that reduced miR-603 expression leads to increased eEF2K expression in TNBC cells, providing the first evidence regarding the mechanistic-basis of eEF2K overexpression in TNBC. Considering that eEF2K contributes to poor patient survival and prognosis (Figure 1C) and plays a cytoprotective role in response to treatments that induce energy stress and cytotoxic effects through induction of autophagy [37] and promotes cell proliferation, cell cycle progression, drug resistance and invasion, reduced or loss of miR-603 seems to be a critical factor leading to induction of eEF2K. Furthermore, our analysis of TCGA databases also indicated that miR-603 tumor suppressor is undetectable in majority of breast cancer samples, preventing us to perform Kaplan-Meier survival analysis with regard to overall survival analysis, suggesting that miR-603 is frequently dysregulated in breast tumors.

Recently, reduced miR-603 has been implicated in malignant transformation of thyroid cancer cells in response to high mobility group A1 (HMGA1) expression [35]. Interestingly, HMGA1 has been proposed to be a master regulator of tumor progression in TNBC by providing oncogenic signaling and metastatic phenotype, EMT and reprogramming TNBC cells into cancer-stem cell like state as well as poor prognosis in breast cancer patients [38, 39]. Thus, future studies are needed to determine if the HMGA1 plays a role in miR603/eEF2K axis and HMGA1 expression leads to downregulation of miR603 and upregulation of eEF2K expression.

**Figure 7:**
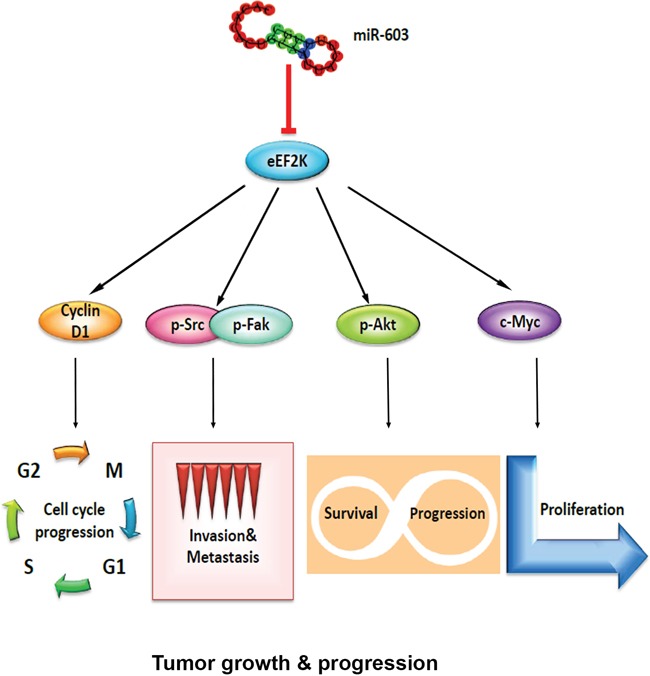
Schematic model of the regulatory pathways involving miR-603 and eEF2K in triple-negative breast cancer

eEF2K is involved in inducing effects on multiple signaling pathways, including the PI3K/Akt, c-myc, and Src/Fak to promote a number of processes associated with migration/invasion (Figure 5). The results of the current study indicate that *in vitro* and *in vivo* overexpression of miR-603 recapitulates the effects of eEF2K suppression in TNBC, including inhibition of tumor cell proliferation, migration, invasion and tumor growth. Not surprisingly, the induction of miR-603 expression in TNBC tumors in mice led to inhibition of PI3K/Akt and Src, further indicating that regulation of these pathways by miR-603 is mediated by inhibition of eEF2K. Furthermore, therapeutic targeting of miR-603 by mimic or eEF2K by siRNA-based therapeutics inhibits tumor growth in an orthotopic xenograft model of breast cancer [16], suggesting that strategies targeting miR-603/eEF2K axis may provide broad antitumor effects through inhibition of multiple oncogenic pathways (Figure 7).

eEF2K expression is associated with resistance to frontline chemotherapeutics such as doxorubicin and paclitaxel, and eEF2K knockdown in an *in vivo* orthotopic model of breast cancer suppresses the growth of established breast cancer tumors and sensitizes the tumors to these chemotherapeutics [16], indicating the role of eEF2K expression in patient survival and prognosis. Thus, future studies combining miR-603-based therapy with chemotherapeutics such as paclitaxel or doxorubicin expected to provide significant enhancement in the antitumor efficacy in TNBC models and elucidate the underlying mechanisms regarding why TNBC patients with higher eEF2K expression have poor clinical outcome and shorter survival.

Given that reinstatement of miR-603 expression in TNBC is associated with inhibition of tumor growth and down regulation of eEF2K, strategies targeting eEF2K directly (i.e., siRNA or inhibitors) or indirectly by targeting its regulators such as miR-603 may be a potential therapeutic approach against TNBC. Recently we identified FOXM1 as the first transcription factor that regulates eEF2K expression in TNBC and its inhibition not only reduces eEF2K expression in TNBC also significantly suppresses TNBC tumorigenesis [13], indicating that understanding mechanism of eEF2K regulation and identification of upstream regulators could also provide foundation for development of novel therapeutic strategies for TNBC, which has poor clinical outcome and patient survival rates due to lack of effective molecularly targeted therapies.

Since many biological disorders result from aberrant gene expression or gene mutations, miRNA replacement therapy represents a promising therapeutic approach by targeting genes that are involved in many pathological processes [40]. In fact, miRNAs are being evaluated in clinical trials in the United States as a therapeutic modality against cancer [41]. Because of the potential of miRNA-based therapies, involving reconstitution of tumor suppressor miRNAs or suppression of oncogenic miRNAs [42], to affect multiple targets or pathways, miRNAs have rapidly garnered attention as a novel class of therapeutics that can be used to modulate gene expression or regulate undruggable targets. Increasing evidence from recent studies supports the therapeutic potential of ncRNA such as siRNA and miRNAs against cancer if delivered properly into tumors [41, 43].

In conclusion, our *in vitro* and *in vivo* results indicate that miR-603 acts as a tumor suppressor molecule that can attenuate the proliferation and invasion of TNBC by directly targeting eEF2K expression. Restoration of miR-603 expression appears to suppress various hallmarks of cancer and may be a potential therapeutic approach against TNBC. Therefore miR-603-based therapy is a promising strategy in the treatment of TNBC.

## EXPERIMENTAL PROCEDURES

### Cell lines and cell culture conditions

The human mammary epithelial cell line MCF-10A and TNBC cell lines MDA-MB-436, MDA-MB-231, MDA-MB-468, BT-549, BT-20 and Human Embryonic Kidney 293 (HEK293) cells were purchased from the American Type Culture Collection (Manassas, VA). MDA-MB-436, MDA-MB-231, MDA-MB-468, BT-549, BT-20 and HEK293 cells were cultured in Dulbecco's modified Eagle's medium (DMEM)/F12 supplemented with 10% FBS and a 100-U/ml penicillin-streptomycin solution (Sigma). MCF-10A cells were maintained in a nutrient mixture consisting of DMEM/F12 supplemented with 5% horse serum, epidermal growth factor, hydrocortisone, insulin and cholera toxin. All cultured cells were incubated at 37°C in a water-saturated 95% air–5% CO_2_ atmosphere.

### Expression of eEF2K protein in human breast cancer patient samples and Kaplan-Meier survival analyses

We downloaded RNASeqv2 Level3 data publicly available from the Cancer Genome Atlas Project (TCGA; https://gdc.nci.nih.gov/) for eEF2K in patients with breast adenocarcinoma (BRCA). Overall survival information for 58 Breast cancer patients was retrieved from cbioPortal (http://www.cbioportal.org/) [36]. We performed Cox regression analysis for associations between survival and eEF2K expression levels. The analysis yielded a hazard ratio of 2 (CI (95%)=(1. 21, 3.17), Wald test p-value =0.0398). For cases with high eEF2K (last tertile, 66-100th Percentile of Range) compared to cases with low eEF2K (first tertile, 0 - 33th Percentile of Range). The Kaplan-Meier plots were generated for this dichotomization. The numbers of patients at risk in low and high eEF2K groups at different time points are presented at the bottom of the graph.

### Cell viability and colony formation assays

The proliferation of MDA-MB-436, MDA-MB-231, and BT-20 cells was analyzed using MTS assay [3-(4,5-dimethylthiazol-2-yl)-5-(3-carboxymethoxyphenyl)-2-(4-sulfophenyl)-2H-tetrazolium] as previously described [11, 16]. Briefly, 1 × 10^3^ to 2 × 10^3^cells/well were seeded in 96-well plates. After overnight incubation, the cells were treated with a synthetic RNA oligonucleotide that mimics miR-603 or with a scrambled negative control miRNA (Ambion). Cell viability was determined at 24, 48, and 72 h using 5 mg/ml MTS. Plates were analyzed at 490-nm wavelength in a VMax kinetic enzyme-linked immunosorbent assay microplate reader (Molecular Devices). To determine the effect of miR-603 on colony formation, we used a clonogenic assay. Briefly, single-cell suspensions were generated for each cell line, and 250 cells were seeded into 24-well tissue culture plates. After 24-h incubation, the cells were transfected with either the control miRNA or miR-603 mimic and either eEF2K siRNA or a control siRNA (Sigma) and cultured for 10-14 days. Colonies were stained with crystal violet, and those consisting of at least 50 cells were counted. Each experiment was performed independently in triplicate.

### Transfections with miRNA mimics and siRNAs

MDA-MB-436, MDA-MB-231, and BT-20 cells were plated in six-well plates at a density of 1.5 × 10^5^ cells/well and transiently transfected with 100 nM mature mimics of miR-603, miR-3613-3p, and miR-3163 or 100 nM control miRNA (all from Ambion) by using HiPerFect transfection reagent (Qiagen) in Opti-MEM Reduced Serum Medium (Life Technologies) according to the manufacturer's protocol. Cells were transfected with 50 nM eEF2K siRNA or control siRNA according to the manufacturer's recommended protocol (Qiagen). After 6 h of transfection, cells were kept in a culture medium containing 10% FBS for up to 72 h.

### Overexpression of eEF2K- To establish stable overexpressed eEF2K cell line

MDA-MB-231 cells were infected with the lentiviral plasmids containing the specified lentiviral vector for eEF2K (NM_013302.3) with CMV promoter (LPP-U0633-Lv105) or the mock vector (LPP-NEG-Lv103) according to manufacturer's recommended protocol. Briefly, MDA-MB-231 cells were seeded into 96-well plate (1 × 10^3^ cells/well) and incubated overnight. Next day, lentiviral particles were diluted in medium containing 5% FBS and penicillin/streptomycin, supplemented by polybrene (EMD Millipore Corporation, Billerica, MA, USA) at final concentration of 8 μg/ml and added into the wells. After 48 h incubation, media were replaced with the puromycin containing media (10 μg/ml) for selection during 3 weeks. (Invitrogen/Life Technologies, Carlsbad, CA). eEF2K gene expression was determined and verified by Western blotting.

### RNA extraction and miRNA and mRNA reverse transcription and qPCR analyses

Total RNA including miRNAs was extracted using the miRNeasy Mini Kit (Qiagen) according to the manufacturer's recommended protocol. The concentration and purity of the RNA were measured by UV absorbance at 260 and 280 nm using an Epoch microplate spectrophotometer (BioTek Instruments). One microgram of RNA was used as a template. Isolated RNA samples were converted to complementary DNA (cDNA) using the qScript microRNA cDNA Synthesis Kit (Quanta BioSciences) under the following conditions: 37°C for 60 min, 70°C for 5 min, 42°C for 20 min, and 85°C for 5 min. miR-603 expression was measured with the PerfeCTa microRNA Assay Kit (Quanta BioSciences) using miRNA primers from Quanta BioSciences using Real-time PCR (qPCR). The miR-603 expression level was normalized to the level of U6 small nuclear RNA (RNU6; Quanta BioSciences), which was used as an endogenous control.

For eEF2K gene expression, reverse transcription (RT) was performed with a RevertAid First Strand cDNA Synthesis Kit (Thermo Scientific) at 42°C for 60 min and 70°C for 5 min. cDNA samples were stored at -80°C until analysis. eEF2K gene expression was measured with theiQ SYBR Green Supermix qPCR Kit (Bio-Rad). The sequences of the sense and anti-sense eEF2K primers were 5’-GGA GAG AGT CGA AGG TCA CG-3’ and 5’-GCA ATC AGC CAA GAC CAT CT-3’, respectively. The sequences of the sense and anti-sense GAPDH primers were 5’-CAA GGT CAT CCA TGA CAA CTT TG-3’ and 5’-GTC CAC CAC CCT GTT GCT GTA G-3’, respectively. cDNA synthesis was verified by detection of the GAPDH transcript, which was used as an internal control. Relative differences in expression were determined using the comparative threshold cycle (2^-ΔΔCt^) method.

### Protein extraction and western blotting

Seventy-two hours after miRNA transfection, cells were lysed in lysis buffer containing protease and phosphatase inhibitors. Lysates were centrifuged at 13,000 × *g* for 20 min at 4°C, and supernatants were collected. The total protein concentration for each sample was determined using the Pierce BCA protein assay kit (Thermo Scientific). Forty micrograms of total protein from each sample was subjected to SDS-PAGE with a 4% to 15% gradient for protein separation and electro-transferred to polyvinylidene difluoride membranes. The expression levels of selected proteins were detected by using specific antibodies for eEF2K, p-EF2 (Thr56), Src, p-Src ( Tyr416),(Cell Signaling Technology), FAK, p-FAK (Try397) (Thermo), p-AKT (Ser473), AKT, cyclin D1 (Santa Cruz) and β-actin (Sigma) and the corresponding HRP-conjugated secondary antibodies [16]. Immunoblots were visualized using HyGLO Chemiluminescent HRP Antibody Detection Reagent (Denville Scientific) in a FluorChem 8900 imager and quantified with a densitometer using AlphaImager software (Alpha Innotech). All experiments were independently repeated three times.

### Reverse phase protein array (RPPA)

RPPA analysis was performed at the Functional Proteomics RPPA Core Facility of The University of Texas MD Anderson Cancer Center. Briefly, MDA-MB-231 cells (2.5 × 10^6^ cells/well) were seeded in six-well plates and transfected with 100 nM miR-603 mimic or control miRNA for 72 h. Cells were washed twice with PBS, and then 100 μl of lysis buffer containing 1% Triton X-100, 50 mM HEPES, pH 7.4, 150 mM NaCl, 1.5 mM MgCl_2_, 1 mM EDTA, 100 mM NaF, 10 mM sodium pyrophosphate, 1 mM Na_3_VO_4_, 10% glycerol, and protease and phosphatase inhibitors (Roche Applied Science) was added to the plate on ice. Cells were scraped and centrifuged at 14,000 rpm for 10 min at 4°C. Supernatants were collected, and total proteins were quantified using the Pierce BCA protein assay kit. The concentration of proteins was adjusted to 1.0 μg/μl. A 4× SDS sample buffer (40% glycerol, 8% SDS, 0.25 M Tris-HCl, 10% 2-mercaptoethanol, pH 6.8) was then added to cell lysates. Protein samples were denatured and stored at –80°C until RPPA processing.

### Cell motility, migration and invasion assays

An *in vitro* wound healing assay was used to measure cell motility and migration. MDA-MB-436, MDA MB-231, and BT-20 cells were plated in six-well plates (2 × 10^5^ cells/well) and cultured in medium containing 10% FBS. After 24-h incubation, the cells were transfected with the control miRNA or the miR-603 mimic. A straight scratch was made on the cell layer using a 200-μl sterile pipette tip (time 0) and the medium was replaced with fresh medium. After the treatments, the cells were photographed using a phase contrast microscope (Nikon Eclipse TE-200-U) to determine the wound width at 0 h. The cultures were continued, and images were captured at 0 and 36h instead of 12, 24, and 48h. Wound healing was measured as the distance migrated by the leading edge of the wound at each time point. The experiments were performed in triplicate.

The invasion assay was performed using transwell inserts coated with matrigel matrix (both from Corning). After 72-h transfection with miR-603, control miRNA, eEF2K siRNA, or control siRNA, 4 × 10^4^ cells in serum-free medium were added to the upper chamber, allowing invasion of the lower chamber containing medium with 10% FBS for 24 h in an incubator. At the end of the invasion assay, invading cells were fixed and stained with Hema 3 (Thermo Scientific), and the cells in the upper chamber were removed by wiping with a cotton swab. Invading cells were counted using a light microscope. All experiments were performed in triplicate, and cells were counted in at least five different fields in each experiment.

### Luciferase reporter assay for miR-603 expression

pEZX-MT06 miRNA reporter vectors containing three different binding sites for miR-603 in the eEF2K 3’-UTR in combination with the luciferase gene were used (GeneCopoeia). pEZX-MT06 miRNA reporter vector containing one point mutation (CACTGCC->TATGACT) was also used. Twenty-four hours before transfection, 1 × 10^3^ HEK293 cells were plated in each well of a 96-well plate. After 24 h, the cells were transfected with the pEZX-MT06 vector (1 μg) together with 50 nM miR-603 mimic or control miRNA. Luciferase activity was measured 48 h after transfection by the Luc-Pair miR Luciferase Assay (GeneCopoeia). For each sample, firefly luciferase activity was normalized to Renilla luciferase activity.

### Preparation of miRNA nanoparticles

For *in vivo* delivery, miRNA was incorporated into pegylated liposomes composed of dimyristoyl-sn-glycero-3-phosphocholine (DMPC) and pegylated distearoyl-phosphatidylethanolamine (DSPE-PEG-2000) (Avanti Lipids). DMPC and DSPE-PEG2000 were mixed at a 10:1 ratio, and miRNA (control or miR-603 mimic, 8ug miR/mouse) was mixed at a 10:1 (w/w) ratio of lipids to oligonucleotides in the presence of excess tertiary butanol. Prior to *in vivo* administration, lyophilized lipid/miRNA complex was reconstituted in 0.9% saline. The liposomal suspension filtered and centrifuged. The liposomes trapped in the filter was reconstituted and used for injections.

### Orthotopic xenograft tumor model

Athymic female nude mice (4-5 weeks old) were obtained from the Department of Experimental Radiation Oncology, MD Anderson Cancer Center. All studies were conducted according to an experimental protocol approved by the MD Anderson Institutional Animal Care and Use Committee. TNBC cells (MDA-MB-436) (2 × 10^6^ in 20% matrigel) were injected into the mammary fat pad of each mouse. Two weeks after injection, when the tumor size reached about 3-5 mm, liposomal-miRNA treatment was initiated. Each mouse received miR-603 or control miRNA (0.3 -mg/kg equivalent of 8 μg/mouse once a week) in a volume of 100 μl for 4 weeks (total of four i.v. injections) through the tail vein. Tumor volumes were measured every week using an electronic caliper. After completion of treatment, mice were euthanized with CO_2_ and weighed to measure tumor growth. Tumor tissues were removed and analyzed by Western blot, immunohistochemistry and TUNEL (terminal deoxynucleotidyl transferase–mediated dUTP nick end labeling) analysis.

### Immunohistochemistry

Tumor tissues were collected at the indicated time points from miR-603-treated and control mice. Tissue sections (5 μm) were stained with hematoxylin and eosin. Immunostaining for Ki-67 and CD31 was performed to evaluate cell proliferation and angiogenesis, respectively, in formalin-fixed paraffin-embedded tumor tissues according to the manufacturer's guidelines. Briefly, after slides were deparaffinized and dehydrated, they were incubated in antigen retrieval solution (Dako) at 95°C for 40 min. After blocking of endogenous peroxidases with methanol containing 3% hydrogen peroxide for 15 min, the tissue sections were incubated with primary antibody for Ki-67 or CD31 at 4°C overnight. The sections were incubated with secondary antibodies at room temperature for 1 h. Afterward, the sections were counterstained with hematoxylin for 30 s and analyzed by microscopy (Nikon Eclipse TE-200-U).

### TUNEL assay

A TUNEL assay (Promega) was used to detect and quantitate apoptotic cells in tumor tissues by measuring nuclear DNA fragmentation according to the manufacturer's recommended protocol. Briefly, MDA-MB-436 tumor sections from control and miR-603-treated mice were incubated with biotin-dUTP and terminal deoxynucleotidyl transferase for 1 h; fluorescein-conjugated avidin was then added, and the tissue sections were incubated for 30 min in the dark. The slides were then stained with Hoechst 33342 dye (Thermo Scientific) to counterstain DNA. Positively stained fluorescein-labeled and Hoechst 33342-counterstained cells were examined under an inverted fluorescence microscope. The apoptosis rate in tumor tissues was obtained by determining the average number of TUNEL-positive cells in five fields in each section.

### Statistical analyses

Data were expressed as means ± standard deviations (SDs). Analysis of variance was used to compare the control and treatment groups. All values were analyzed using the two-tailed Student t-test. *p*-values < 0.05 were considered statistically significant. Analyses were performed using GraphPad Prism (version 6.02) software.

## SUPPLEMENTARY MATERIALS FIGURES


