# Correction: MicroRNA 603 acts as a tumor suppressor and inhibits triple-negative breast cancer tumorigenesis by targeting elongation factor 2 kinase

**DOI:** 10.18632/oncotarget.28533

**Published:** 2023-10-31

**Authors:** Recep Bayraktar, Martin Pichler, Pinar Kanlikilicer, Cristina Ivan, Emine Bayraktar, Nermin Kahraman, Burcu Aslan, Serpil Oguztuzun, Mustafa Ulasli, Ahmet Arslan, George Calin, Gabriel Lopez-Berestein, Bulent Ozpolat

**Affiliations:** ^1^Department of Experimental Therapeutics, The University of Texas MD Anderson Cancer Center, Houston, Texas, USA; ^2^Department of Medical Biology, School of Medicine, Gaziantep University, Gaziantep, Turkey; ^3^Department of Biology, Kirikkale University, Kirikkale, Turkey; ^4^Center for RNA Interference and Non-Coding RNAs, The University of Texas MD Anderson Cancer Center, Houston, Texas, USA

**This article has been corrected:** In Figure 4A, the MDA-MB-436 cell images are accidental duplicates of the MDA-MB-231 images. The corrected Figure 4, produced using the original data, is shown below. The authors declare that these corrections do not change the results or conclusions of this paper.

Original article: Oncotarget. 2017; 8:11641–11658. 11641-11658. https://doi.org/10.18632/oncotarget.14264

**Figure 4:**
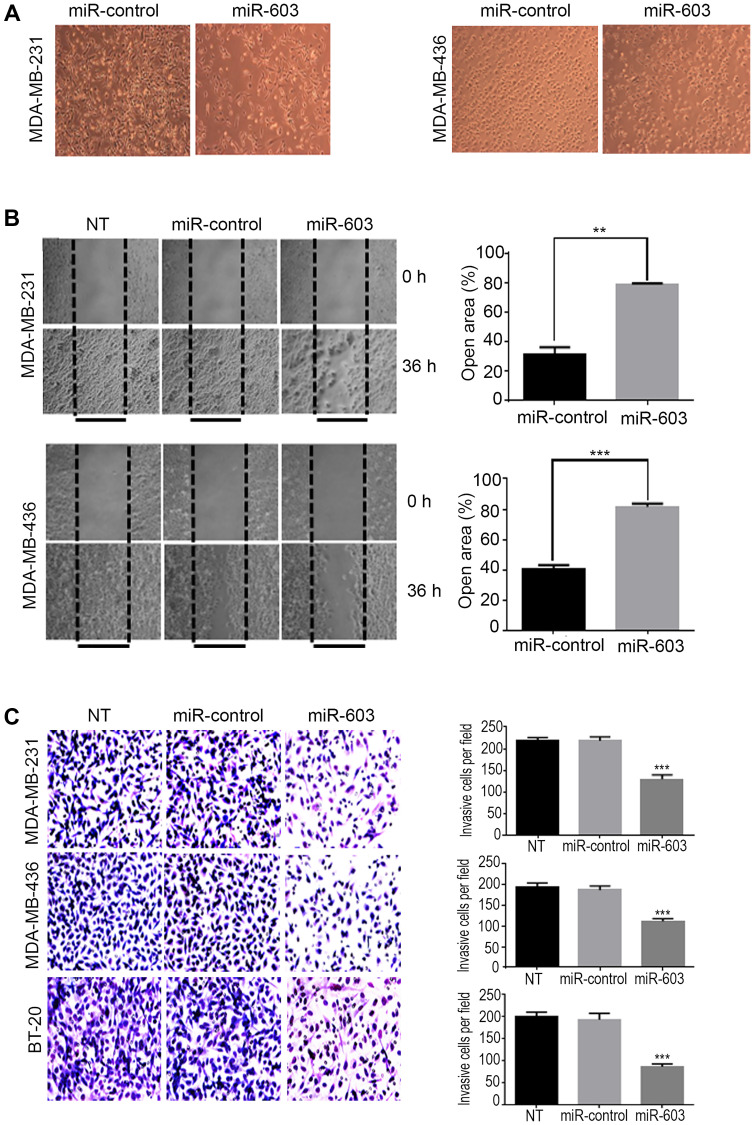
Transfection of TNBC cells with miR-603 suppresses migration and invasion of the cells *in vitro*. (**A**) Morphological changes in MDA-MB-231 and MDA-MB-436 cells after 48-h transfection with 50 nM miR-603 or control miRNA. Representative phase contrast micrographs are shown. (**B**) MDA-MB-231 and MDA-MB-436 cell lines that were transfected with miR-603, or miR-control or that did not undergo transfection (NT) were assessed for migration with the wound healing assay. After 72-h transfection, a wound was formed by scraping, and the area of the wound was measured at 0 and 36 h. The relative percentages of wound closure per field are shown on the right as means ± SDs. (**C**) the invasiveness of MDA-MB-231, MDA-MB-436 and BT-20 cells was assessed by using a matrigel transwell assay. The cells were transfected with miR-603 or miR-control or not treated (NT). After 72-h transfection, the cells were transferred to transwell chambers and incubated for 24 h. The invading cells were counted, and mean ± SDs from triplicate experiments are shown on the right (^***^ *p* < 0.001).

