# Supplementary material for: MicroRNA 603 acts as a tumor suppressor and inhibits triple-negative breast cancer tumorigenesis by targeting elongation factor 2 kinase
Source: Oncotarget. 2016 Dec 27;8(7):11641–58. doi: 10.18632/oncotarget.14264 (PMC5355293; doi:10.18632/oncotarget.14264)
Supplement: Supplementary file 1 [file oncotarget-08-11641-s001.pdf]

MicroRNA 603 acts as a tumor suppressor and inhibits triple-negative breast cancer tumorigenesis by targeting elongation factor 2 kinase

SUPPLEMENTARY FIGURES

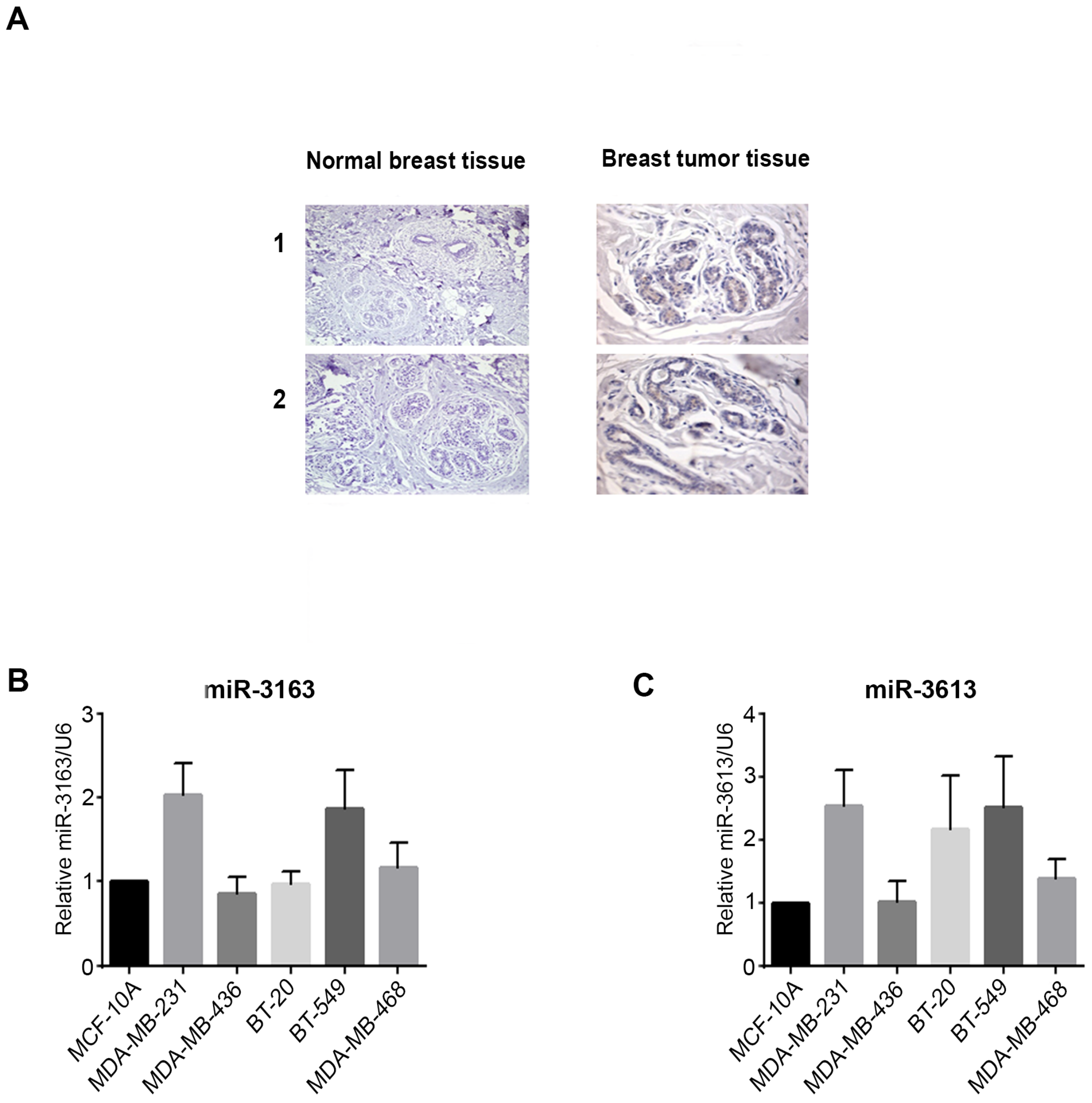

Supplementary Figure 1. A. The protein expression levels of eEF2K in patient tumor tissues and adjacent normal tissues were determined by immunohistochemistry. Relative expression levels of miR-3163 **B.** and miR-3613 **C.** in TNBC cell lines and in MCF-10A cells were quantified by qPCR using specific primers. The data were normalized to the expression of U6 small nuclear RNA and are shown as means with SDs for three independent experiments.

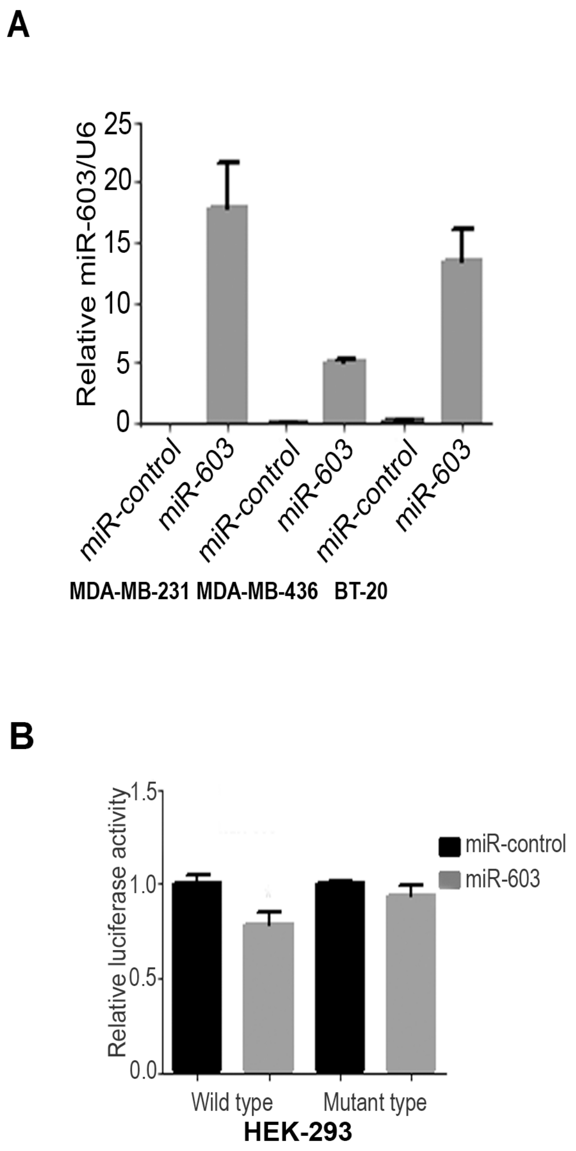

Supplementary Figure 2. A. The expression of miR-603 after transfection with the miR-603 mimic or the scrambled control miRNA (miR-control) in MDA-MB-231, MDA-MB-436, and BT-20 cells was validated with qPCR. **B.** Luciferase reporter assay showed that miR-603 directly targets the eEF2K 3'-UTR-luciferase reporter (wild type or mutant miR-603 binding sides)

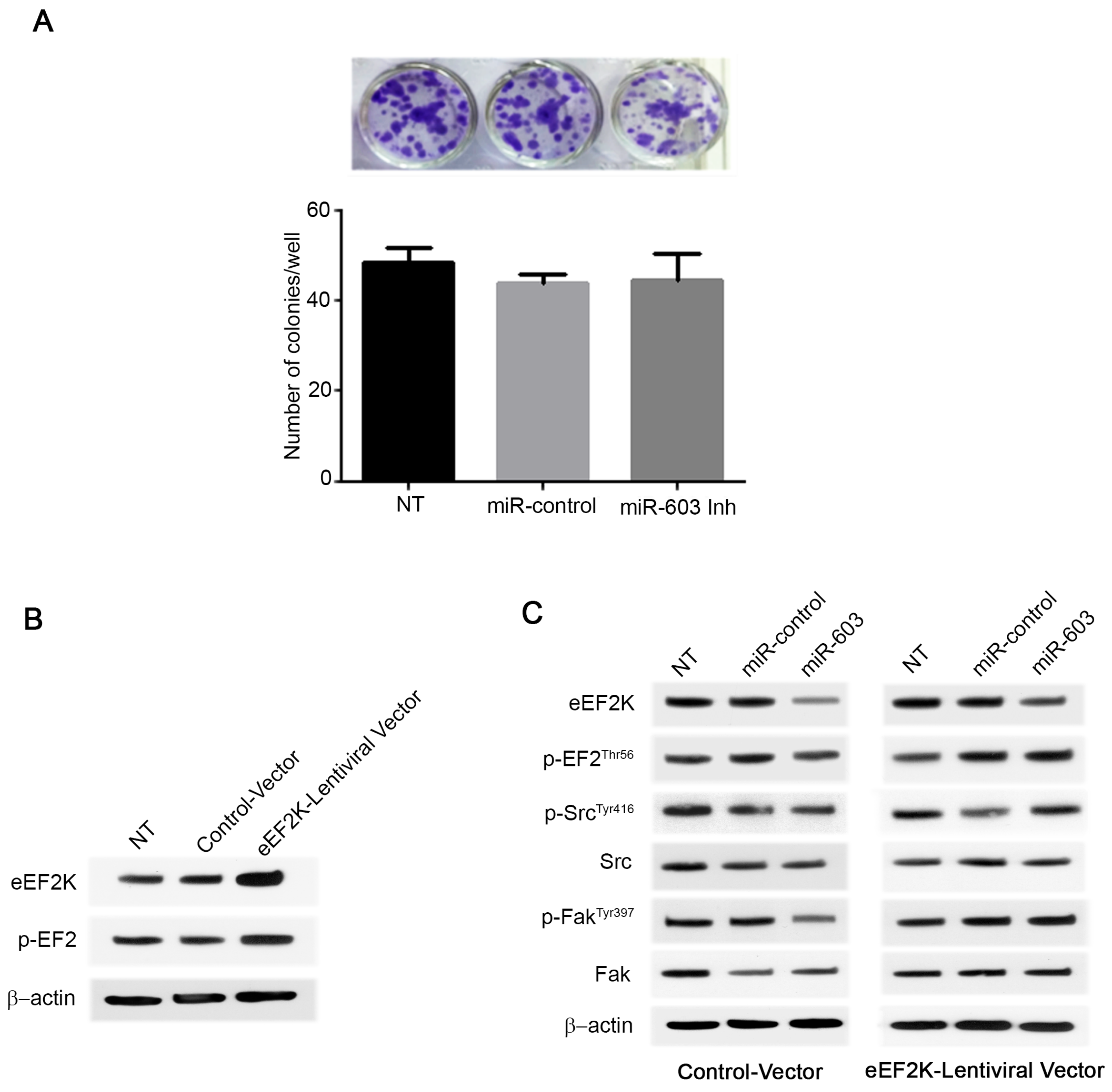

Supplementary Figure 3. A. Inhibition of miR-603 had no affect on clonogenic ability of MDA-MB-231 cells by using miR-603 inhibitor. **B.** Western blot analysis of eEF2K and p-EF2 ^{Thr56} after eEF2K gene over-expression using lentiviral system. **C.** Western blot analysis of eEF2K, p-EF2 ^{Thr56}, p-Src ^{Tyr416}, total Src, p-Fak^{Tyr397} and total Fak, in eEF2K overexpressing or control MDA-MB-231 cells. β-Actin was used as a loading control.
